# Patient- and Community-Oriented Primary Care Approaches for Health in Rural, Remote and Resource-Dependent Places: Insights for Eco-Social Praxis

**DOI:** 10.3389/fpubh.2022.867397

**Published:** 2022-05-26

**Authors:** Chris G. Buse, Sandra Allison, Donald C. Cole, Raina Fumerton, Margot Winifred Parkes, Robert F. Woollard

**Affiliations:** ^1^Centre for Environmental Assessment Research, University of British Columbia (Okanagan Campus), Kelowna, BC, Canada; ^2^Vancouver Island Health Authority, Victoria, BC, Canada; ^3^Dalla Lana School of Public Health, University of Toronto, Toronto, ON, Canada; ^4^Northern Health, Terrace, BC, Canada; ^5^School of Health Sciences, University of Northern British Columbia, Prince George, BC, Canada; ^6^Department of Family Practice, Faculty of Medicine, University of British Columbia, Vancouver, BC, Canada

**Keywords:** patient engagement, community engagement, public health, primary care, rural health, resource development

## Abstract

Accelerating ecological and societal changes require re-imagining the role of primary care and public health to address eco-social concerns in rural and remote places. In this narrative review, we searched literatures on: community-oriented primary care, patient-oriented research engagement, public health and primary care synergies, and primary care addressing social determinants of health. Our analysis was guided by questions oriented to utility for addressing concerns of social-ecological systems in rural, remote contexts characterized by a high degree of reliance on resource extraction and development (e.g., forestry, mining, oil and gas, fisheries, agriculture, ranching and/or renewables). We describe a range of useful frameworks, processes and tools that are oriented toward bolstering the resilience and engagement of both primary care and public health, though few explicitly incorporated considerations of eco-social approaches to health or broader eco-social context(s). In synthesizing the existing evidence base for integration between primary care and public health, the results signal that for community-oriented primary care and related frameworks to be useful in rural and remote community settings, practitioners are required to grapple with complexity, durable relationships, sustainable resources, holistic approaches to clinician training, Indigenous perspectives, and governance.

## Highlights

- Multiple frameworks within the patient-oriented care movement align with principles of eco-social approaches to health.
- Attention to processes of patient and community engagement are crucial for incorporating eco-social approaches to health in primary and public health collaboration.
- Multi-disciplinary teams and other sector organizations can support use of tools identified in this review.
- Frameworks, processes and tools relevant to treating both patients and ‘communities of patients' need adaptation for application in rural areas.

## Introduction

Rural and remote places are often defined by their physical isolation from urban centers, strong community connectedness, a high degree of reliance on natural resource extraction (e.g., forestry, mining, oil and gas, agriculture, fisheries, ranching, renewables) to support local livelihoods, and lower levels of access to key services, especially those in the healthcare sector (1). When overlaid with the complex array of impacts from climate change, the foundational contextual components of rural and remote places create unique impacts on the health of rural populations (2), with consequent implications for the health sector (3) and worker training (4). This has necessitated the need for new framing for the role of “environments of health and care” (5) and creative responses in partnership approaches to integrated health care reform that focus attention on upstream causes (6, 7).

However, unpacking “environments of health and care” in rural, remote and resource dependent places requires consideration of not only the broader ecological and social contexts in which health systems operate, but also the complex pathways by which community members become patients due to injury or illness. In this contribution, we review multiple approaches to primary care practice that may enable more engagement with the determinants of health, and merge this literature with eco-social considerations that can enable researchers and practitioners to be more attentive to the community and geographic contexts which influence human health. In other words, this paper seeks to shed light on approaches that can incorporate understanding of health in rural communities where these places have characteristics of social-ecological systems [i.e., reflecting interdependent relationships between the social actors and institutions embedded within a biophysical system; (8)]. We set out to review relevant patient-oriented and community-oriented approaches, and revisit primary care and public health collaboration literatures, and viewing them through an eco-social lens. Specifically, we posed the following question:

What existing evidence can help health teams better ally with communities in understanding and addressing eco-social pressures relevant for health in rural and remote communities?

Informed by the literature reviewed in this introduction, we begin by characterizing the challenges posed to human health in rural and remote settings and introduce the concept of “eco-social” research and practice. We then provide an overview of our review methods, and go on to present the resources identified in terms of relevant frameworks, processes and. tools identified in the literature reviewed. This leads to an exploration of the implications, challenges, opportunities and questions that warrant ongoing attention in order to support health teams, practices and policies to better reflect the eco-social context for health in the rural and remote communities they serve. Our analysis underscores six areas of innovation emerging in Canada and beyond, including Indigenous leadership and other integrative approaches to health that reflect the nuances of rural and remote contexts.

### The Challenges of Health in Rural, Remote and Resource-Dependent Contexts: Introducing the Eco-Social Approach

The challenges of reflecting the complex health context for rural and remote communities are a topic of long-standing interest among health researchers and policy makers. For example, some literatures focus on the broader political economic drivers of rural and remote resource development as a key health determinant, highlighting the role of extractivism inexorably shaping conditions for health, via multiple intrusive pathways ranging from toxicological exposures to changes in the determinants of health (9–11). In grappling with the complexity of rurality, others have focused on the importance of social relationships in isolated places—often framed as “context”—particularly for rural mental health (12). Moreover, Bourke et al. (1) developed a framework highlighting the dynamic interplay between societal structures and individual agency to conceptualize the multi-layered, diverse components involved in rural health. Less common have been explorations of rurality and physical environments in determining the health of populations in rural places (13, 14). The disproportionate impact of wildfires on rural communities (15) and cumulative adversity impacts on mental health in rural settings (16) serve as exemplars of the interlinkages between social and ecological systems in rural and remote places, and how they shape health outcomes.

Accelerating ecological, physical, environmental, and societal changes have prompted an urgent need to better reflect the combined ecological and social context for health in rural and remote places. A narrative review of research priorities for rural and remote primary health care included responding to climate change as an important priority (17). Similarly public health actors have focused on the important role of ecological determinants of health (18) to complement the social determinants of health, as part of an expanding array of approaches linking ecosystems, environments and health (19, 20). In other words, ecological health can be thought of as the overall health of the biome, which includes humans and all other species, and “eco-social” as a framing of the dynamic interactions between ecological and social contexts and their role in shaping health status (21). Eco-social focuses “attention on the reciprocity among the ecological and the social as essential features of a proactive orientation to future health and collective well-being, especially in the face of rapid planetary-scale ecological changes that threaten human well-being and societal stability” [(22), p 61]. The framing of “eco-social” here is therefore strongly related to early conceptualizations of “ecosocial” by Krieger (23) who used the term intentionally to reflect the social production of health and illness across multiple scales. However, our use of “eco-social” is intended to reclaim the “eco” to be more overtly ecological, which has been markedly absent from the “ecosocial” literature (24), despite Krieger (25) later expanding this orientation to better reflect ecological and biological contributions to health.

As a result of this thinking, health impact assessments of resource extraction and development proposals in rural and remote communities—as one tool to understand the health impacts of major projects which have historically been focused primarily on physical environment determinants, such as contamination of air, water or soil—have been broadened to incorporate social determinants (26) and health equity analyses (27). Broadening the time horizon, the cumulative environmental, community and health impacts of multiple resource development projects have been examined by integrated natural, social and health science teams working collaboratively (14) across all the associated challenges (20).

Eco-social approaches to health acknowledge that within many rural and remote community catchments (in the ecological watershed sense as well as “service-provided” sense) a combination of agriculture, forestry, mining and fracking (among others) all occur on a single land base. These activities generate complex interrelated sets of benefits and costs for the livelihoods, lifestyles and life-choices of the individuals and the communities in which they occur. Both public health and primary care providers may recognize the combined ecological and social influences on the lives of their populations and patients. Yet little guidance is available, beyond generic health promotion approaches, on how to work with both individual patients and communities (as “collectives” of patients) to promote health, in ways that match the complexity of the eco-social concerns which rural and remote communities face. In light of this, what frameworks exist to support health systems engage with the complexity of social-ecological systems, and how can eco-social thinking help?

## Research Methods

We reviewed approaches deemed relevant by the authors based upon professional, academic and personal experience. We explored opportunities and interactions among the following four broad literatures, focused primarily on the role of primary care providers:

- an organizational focus on *community-oriented primary care*, one of the earliest approaches to engaging with patient contexts and communities (28);
- the more recent *patient-oriented research and practice* approaches, fostered in Canada by national health research funders (see Strategy for Patient-Oriented Research or SPOR https://ossu.ca/about-us/what-is-spor/). Patient-oriented research and practice is about engaging patients to improve the delivery of high-quality, appropriate and cost-effective care in ways that can situate patients in the context of their communities and broader life trajectories;
- approaches explicitly addressing *collaboration among primary care, public health and health systems*, in the integration of engaged communities to create healthy environments [(29), see Table 1]; and
- engagement of *primary care with social determinants of health* (41) as an approach which potential to be expanded to strengthen appreciation of eco-social approaches to health (22).

**Table 1:** Assessment of potentially most relevant frameworks in relation to reflective questions.

First Author(s) [date(s)]^*^–article type	**Framework**	**Includes diverse patient life trajectories, mobility, vulnerabilities and assets?**	**Indicators tapping eco-social contexts for health?**	**State of application?^†^**	**Aspects applicable to practices-communities in diverse rural, resource development regions?**	**Challenges uncovered/addressed for operationalizing?**
Gofin and Gofin (30)—review, Gofin and Foz (31)—Catalonia	Community-Oriented Primary Care (COPC)	Mostly, yes	In some applications, particularly in lower and middle income countries, and rural areas	Decades of institutionalization	Some rural applications demonstrated overall framework with multiple steps.	Most successful COPC undertakings have been externally funded and associated with academic institutions (28)
Blumenthal (32)—review and institutional case study	Clinical Community Health	Mostly, yes	No	Promising approach to institutionalization	Varies across particular applications referenced.	Resources to maintain fidelity with the model—Teams, staff, skills, commitment, dedication, time, patience
Bourke et al. (1)—conceptual with two applications	Comprehensive conceptual framework for the analysis of rural and remote health situations	Unclear	Yes	Application in several Australian (33) and other places	Yes, rural in both the primary care reorganization and Aboriginal health promotion applications.	Multiple levels of power and need for negotiation discussed in each of two examples
Bodenheimer and Sinsky (34)—conceptual	Triple and Quadruple Aim	In patient-centeredness	Some applications e.g., Miranda et al. (35)	Promising approach	Elaborated in some applications.	Not addressed
Tipireni et al. (36)—review with case studies	Accountable Communities for Health	Unclear	Yes, in one case study	Empirical evaluation	Unclear extent to which applicable in resource development regions.	Not addressed
Pelletier et al. (37)—case study	Patient partnership in knowledge translation	Yes for those with serious mental illness	No	Promising approach	Urban example, but involvement of patients and families in multiple ways exemplary.	Additional supports needed for active involvement of patients with serious mental illness
Woollard et al. (38)—conceptual	Social accountability	Yes	Not explicitly	Promising approach	Yes, though not explicitly articulated.	Generic
Holroyd-Leduc et al. (39)—case study with review elements	Patient engagement (1rly in research)	Certainly vulnerabilities (focused on frail elderly) and assets	Broadly considered	Promising approach and ethical imperative	Approach used with combination of evidence, face to face and virtual discussions.	Numerous discussed, particularly power differentials, accessibility with multiple suggestions for addressing them
Orkin (40)—review (with descriptive appendix of studies)	Clinical Population Medicine	Varies by application, [see Appendix]	No	Varied, but argue that lots of examples of application	Some potential tools identified (see below).	Generic in this review

* *Chronological*.

† *State of Application categories: interesting idea, promising approach, empirical evaluation, decades of institutionalization*.

The timing of our research created some thematic constraints within our review. One example is that the planetary health literature, with a focus on patients and/or communities in rural places, was not extensive within the timeframe of our searches. Likewise, ongoing expansion of Indigenous-led literatures profiling contextually relevant approaches to Indigenous health has far-reaching relevance to an array of rural and remote contexts. Although these literatures were not a main focus of our review, we do introduce literature known through authors' engagement and familiarity in these areas in the final section of discussion and implications.

### Searches, Yield and Relevance

Search terms and methods of the English language scholarly literature searches included four targeted areas of literature:

1. Community-oriented primary care (COPC) and its analogs in Medline and EMBASE were searched through the dates 1980–2018. Additional searches were performed of select websites, and using Google Scholar with follow-up of key references. Among the 1,189 articles, 206 named COPC in the abstract. Abstract review of 100 full articles found 50 sufficiently relevant to the integration of public health and primary care. The majority of the latter (64% *n* = 32) were narrative pieces, with some encouraging evaluative work spanning decades.
2. Patient-oriented research and engagement in PubMed, Ovid MEDLINE, CINAHL Complete and Biomed Central from 2009 to 18. Among 376 articles identified, 244 were duplicates, and 44 articles were deemed sufficiently relevant for full-text review. An additional 40 were identified through review of reference lists of these articles (total 84). Perhaps understandably, articles tended to focus on discrete communities of patients dealing with specific health outcomes. The majority (69%, *n* = 58) were in secondary or tertiary care settings, less potentially applicable to primary care.
3. Environment and ecological in public and community health and primary care through Google Scholar without date limits. We primarily relied on scoping reviews (42, 43) and analyses conducted over the last decade (40).
4. Social determinants and primary care through Google Scholar without date limits where we again relied primarily on identified review papers (44).

Article titles and abstracts were screened primarily for relevance to our research question and rural and remote places within the context of resource development activities. For relevance judgements, we drew on the research, policy, practice, training, organizational and lived experience of the authors and research assistants (see acknowledgments). We are all settlers, but live/work or have lived/worked in and with rural and remote communities in Canada and internationally for decades, as primary care providers and clinician consultants, and as public health staff and leaders. We are also researcher-mentor-academics engaged with partners at multiple jurisdictional levels (38).

### Analysis

Given the broad array of potentially relevant literature, and the plethora of frameworks, processes and tools encountered, the authors used a series of reflective questions to help focus our analysis. These included:

1. Do frameworks, processes and tools identified pick up on diverse patient life trajectories, mobility, vulnerabilities, and assets?
2. Do they explicitly include indicators tapping eco-social concerns for health?
3. What is the state of application of each (ranging from interesting idea, through promising approach, empirical evaluation, to decades of institutionalization)?
4. What aspects of the frameworks, processes and tools could be useful in diverse rural and remote places where resource development is past, occurring or planned?
5. What are the challenges uncovered/addressed for operationalizing the frameworks, processes and tools?
6. What might be gained by more effective collaborations between primary care and public health at the community level?

We used qualitative analysis methods (45) to respond to the questions, organizing our findings on the different resources (frameworks, processes and tools) in tabular form with illustrative examples (See Tables 1–3). We built on the findings of this narrative review (see Resources Uncovered below) through iterative discussion among the authors, resulting in a synthesis of key implications, challenges, opportunities and questions (see action 4).

**Table 2:** Assessment of potentially most relevant processes in relation to reflective questions.

First Author(s) [date(s)]^*^–article type	**Process**	**Includes diverse patient life trajectories, mobility, vulnerabilities and assets?**	**Indicators tapping eco-social contexts for health?**	**State of application?^†^**	**Aspects applicable to practices-communities in diverse rural, resource development regions?**	**Challenges uncovered/addressed for operationalizing?**
Leonhardt et al. (46)—case study	Community-based patient advisory council	Focus was medication use safety, not patient distinguished	Not included	Promising approach	Rural county with multiple health centers, so likely applicable.	Health provider involvement, creating trust and respect, time-intensive for personnel involved.
Tisnado et al. (47)—case study	Community-partnered research—CBPR	Of participating community researchers	Not focus	Demonstration project process documentation	Cultural group rather than geographically defined. Working through different values, establishing mechanisms for interaction between community members and providers- researchers all instructive.	Time availability, preferred communication modes, data sharing issues, limited funding for community partners.
Joosten et al. (48, 49)—multiple case study	Community engagement studios	Yes	Not focus	Demonstration project evaluation	Potential for adapting already developed research ideas. Could be done virtually in rural areas, depending on connectivity.	Core funding support and adequate information to stakeholders needed. Reasonable cost.
Etchegary et al. (50)—case study	Town halls on health research	Not directly, though some shared	Not clear	Promising approach	Rural communities included, could tap health research interests.	Time for planning and use of appropriate language.
Marcus et al. (51) [and Moosa et al. (52)]—multiple case study	(ward-based) Primary care outreach	Vulnerabilities and assets yes	Yes, rurally including water and sanitation	Demonstration project evaluation	Yes, complementary responsibilities in communities with travel to households.	Organizational independence as part of regional health services, with separate staffing and resources.
Kaufman et al. (53)—multiple case study	Health Extension broadly, though distinct models in five different states	In some practices, in some states	Not explicit	Demonstration project evaluations	Several explicitly rural efforts. Experience of building sustained relationships with practices and community coalitions; documenting success in broad terms as well as diverse outcomes of meaning to different stakeholders; understanding that health extension can be carried out by an individual or group depending on resources.	Challenge in USA of market-based health care corporations buying up primary care practices. Need for long-term, sustained fundraising beyond grants.
Shahzad et al. (43)—systematic review	Use clinical opportunities to address underlying causes of health problems	Yes	Built environment—housing in the city (54)	Some empirical evaluation around other kinds of information	Issues addressed in encounter EHR could be eco-social relevant ones e.g., exacerbation of asthma or COPD by wildfires (55).	Generic
	Use clinical encounters and share data (e.g., Electronic Health Records) to build community databases (54, 56)	Potential	Not generally	Some demonstra- tion project evaluation around other kinds of information	Sharing of anonymous, aggregate patient utilization and population information example Bruckner and Barr (57) specifically noted collaborative work in rural county.	Generic
Johnston et al. (58)—case study	Community-engaged health services planning	Subsumed	Only indirectly in effects on transportation	Demonstration project evaluation	All, with a focus on health providers, authorities, systems.	Potential power differential between health providers and other engaged partners.

* *Chronological*.

† *State of Application categories: interesting idea, promising approach, empirical evaluation, decades of institutionalization*.

**Table 3:** Assessment of potentially most relevant tools in relation to reflective questions.

First Author(s) [date(s)]^*^–article type	**Tool**	**Includes diverse patient life trajectories, mobility, vulnerabilities and assets?**	**Indicators tapping eco-social contexts for health?**	**State of application?^†^**	**Aspects applicable to practices-communities in diverse rural, resource development regions?**	**Challenges uncovered/ addressed for operationalizing?**
Mullan et al. (59)—concept and specific application	Geographic retrofitting	Likely	Not yet	Promising approach	Good potential to map patient sources for primary care, emergency utilization, including unincorporated rural areas	Sparseness of census and other data in rural, remote areas
Dulin et al. (60)—case study of application	Geographic information system (GIS) integration and analysis	Yes through Multi Attribute Primary Care Targeting Strategy (MAPCATS)	Not yet	Promising approach	Good potential to map patient sources for primary care, insurance coverage, emergency and hospitalization use, for regions with rural and urban centers	Smaller populations translate into data limitations from nationally representative surveys where small communities may have few people representing an area
Lebrun et al. (61)—multiple case study [also part of COPC literature]	Community health assessment	Likely	In some health centers engaged with environmental justice organizations	Substantial examples, with some empirical evaluation	Included health centers in rural areas. Complemented community health assessment with community needs assessments, ongoing data collection and analysis, use of surveillance data, and program evaluation	Limited integration and interoperability of data sources, within health centers as well as between health centers and partner organizations
Andermann (44)—review	Screening tools as part of patient encounters	Yes, on vulnerabilities	Housing perhaps	Promising approach	Expanding to eco-social contexts for eco-social concerns and impacts as optional template on electronic health records.	Lewis et al. (62) documented the challenges community health center clinicians faced in identifying, treating and accounting/billing for social determinants of health. Included clinician skills and tools, organizational response capacity, and economics of reimbursement. Similarly Gold et al. (63) re: electronic health record integration challenges.
	Analogous to Social Prescribing referrals	Yes, particularly vulnerabilities	Some, as per Young et al. (64)	Interesting idea	Potential for navigator and champion roles in eco-social prescribing e.g., to community member who shares snow shoes with youth and takes them out for walks in woodlands.	Potential challenges due to smaller tax bases, less health and social service capacity in rural areas. Yet also more green space for land-based healing.
Furst et al. (65)—review	Eight mental healthcare ecosystems description/assessment tools	Mostly diagnosis or demographic descriptors	Ecosystem term applied to health care system at different scales but not explicitly eco-social factors	Empirical evaluations	Relevant to mental health services in broad regions, but lack rural specifics	Several challenges in application for health services research

* *Chronological*.

† *State of Application categories: interesting idea, promising approach, empirical evaluation, decades of institutionalization*.

## Results

Our review of literature identified a range of resources potentially relevant to understanding the complex context for health in rural and remote communities. The frameworks, processes, tools presented here reflect terminology and priorities presented by the authors and, in the following section their implications are discussed in relation to contemporary eco-social context for health.

### Frameworks

Our review surfaced a number of frameworks that theorize and describe relationships between primary care and public health in different contexts. Among the 19 named frameworks broadly related to the engagement of patients and communities, a subset of nine seemed most relevant to our overall research question (see Table 1).

Historically, Community-Oriented Primary Care (COPC), and its permutations e.g., Clinical Community Health (32), have been the most prominent. Defined by Mullan (66) as “the continuous process by which primary care is provided to a defined community on the basis of assessed health needs through the planned integration of public health practice with the delivery of primary health care services” applications have occurred globally, including in rural areas (30). In this definition, public health practice was primarily understood as clinical prevention services, particularly in assessment of outcomes. A systematic review (67) observed some evidence of effectiveness in increasing coverage of clinical preventive services and the usefulness of COPC as an educational orientation for primary healthcare providers (32). Mixed evidence was available of use of the COPC framework to understand eco-social concerns.

Improving patient access and outcomes, containing costs and improving population health has been the goal of so-called “Triple Aim” approaches, now expanded to the “Quadruple Aim” in order to include the goal of improving the work life of health care providers, both clinicians and staff (34). Related are Accountable Communities for Health (36) as an implementation of social accountability at the community level [e.g., (38)] with the emphasis on both responsiveness to community needs and appropriate governance structures involving community members. Some examples addressed included environmental determinants of healthy behaviors, although this was not the norm in the literature.

Relatively more emphasis was given to researcher-, provider- and patient-initiated partnership approaches (37) and patient-caretaker engagement strategies (39) which could be applied to address eco-social concerns. Indeed, some consideration, particularly in more rural-specific literatures considers “integrated primary care” to include person- and family-centered primary care which can build trust, while establishing accessible and continuous relationships (68). Categorization of clinical population medicine approaches in primary care (40) has been complemented by conceptualization of bridges across or areas for synergy between clinical care and public health (43). These could support integration of eco-social concerns, though this remains a goal yet to be realized. Among the areas for synergy, two were particularly promising: “identifying and addressing community health problems” and “strengthening health promotion and health protection” (43)—see sections Processes and Tools below for further elaboration.

### Processes

While frameworks can be helpful to situate relationships between public and primary health systems, they can also be opaque as to the processes which underlie moving from an over-arching goal (e.g., more/better collaboration) through to tangible actions that improve patient and population health (69–71). Structured relationships between health care and community organizations have historically been an important part of COPC (e.g., including community members on boards). Although not the focus of this paper, attention to the nature of engagement-collaboration and governance when engaging patients/communities around eco-social issues remains crucial.

For example, Leonhardt et al. (46) reported on a community-based patient advisory council extending their role from patient medication safety to broader safety initiatives in participating communities. Tisnado et al. (47) described community-partnered research with an ethno-cultural community, emphasizing the building of relationships around shared values. Joosten et al. (48) developed a structured approach to systematically engage stakeholders through community engagement studios. This intriguing method for more research-oriented university health sciences groups to obtain feedback on research proposals incurred modest additional costs for the helpful feedback received (49). Etchegary et al.' (50) reached out to rural communities with town halls for both research and healthcare improvement discussions and prioritization. Some consultation with communities could probably be done virtually, as per community hub high risk intervention initiatives (72). A project involving multiple partners in consultations for sustainable rural health care systems found relationships and change over time as core emergent themes in their qualitative research (58).

In the literatures examined, collaborative work involving professionals and community stakeholders to identify and better respond to complex determinants of health appeared to be a necessary condition for incorporating community context, including the recognition of the socioeconomic contexts which create conditions for patients to become “super-utilizers” of healthcare systems (73). For example, most patient oriented literature (*N* = 84) spoke to the need for inter-professional teams including: multi-care team + public health + community members (22%, *N* = 18) and public health + primary care teams (14%, *N* = 11). Although some active clinician participation is needed, many commentators note the importance of resources for non-clinical staff to be included in patient population tracking and linkage to other resources, called “enabling service providers” by Lebrun et al. (61) and others [see, for example, (74, 75)]. Tipirneni et al.' (36) noted the importance of organizational mechanisms at multiple levels for addressing determinants of health. Marcus et al. (51) assessed the strengths and weaknesses of (ward-based) primary care outreach to households and communities not currently accessing primary health care [with (52) similar for an urban- setting]. The functions were filled by both existing health center staff (e.g., nurses, and new staff such as community health workers). Their recommendation was for greater independence, both organizational and budgetary, for such outreach initiatives. Kaufman et al. (53) assessed five state initiatives in different kinds of health extension out of academic health science centers, some in rural areas. Widely different kinds of extension activities occurred involving multiple players in public, private and allied social service and health sectors, some of which explicitly engaged in addressing determinants of health in rural communities.

Turning from outreach to more clinically focused primary care activities, Shahzad et al. (43), recommend “use [of] clinical opportunities to identify and address underlying causes of health problems.” They cite one example which dealt with a more classic environmental cause: housing quality in a city (54). Nevertheless, one can imagine individual electronic health record data generated through templates including a variety of potential eco-social concerns such as:

- Ecological grief (76);
- Repeated adversity (16), including heat events, wildfires and flooding, such as those experienced by rural populations in British Columbia in 2021, with associated mental health-well being impacts;
- Exposure to wildfire smoke exacerbating respiratory conditions (55), particularly where primary care providers also provide emergency services in many rural settings; and
- Connection to the land as an asset to promote health (77), support more robust recovery from disasters (15) or engage in land-based healing (78).

Such examples of opportunities respond to Shahzad et al. (43) recommendation to develop public health and primary care interfaces to “use clinical encounters and share data to build community-wide databases.” Gosling et al. (54) described sharing of anonymous, aggregate primary care patient population information with public health, resource development proponents, social services, and others for program planning and monitoring changes in population health over time. They echoed Calman et al. (56) examples of EHR joint use. Bruckner and Barr (57) provide a strong example of sharing health status and utilization information in a US rural county to address diabetes (though environmental components are underexamined). In contexts characterized by a high degree of reliance on resource extraction and development, one could also imagine using electronic health record data to help identify increased rates of Intimate Partner Violence among populations linked to resource development or the proportion of new pregnancies potentially affected by mutagenic exposures from resource extraction work or waste exposures. Further, crossing sectors, health authorities have linked with wildlife-environmental colleagues reporting networks to address linkages between wildlife and human health (79).

### Tools

Given the presence of frameworks and processes to better link public health and primary care in working with communities, what tools might assist integration of eco-social approaches to health? Table 3 sets out some potential tools for application or extension.

Community health assessment has been a key component of COPC since its inception and was included in Shahzad et al.' (43) review. A good example is the conduct of annual community health needs assessments among US federally-funded health centers in Lebrun et al.' (61) examination of primary care and public health activities. Mullan et al. (59) use of GIS to reflect on patient population dispersion across a county and Dulin et al. (60) work on prioritizing data components and then joint mapping of them could also be useful tools. Unfortunately, population sparseness and geographically large units for analysis in many rural areas pose challenges in achieving precise information. This is in part driven by small populations, but also in part by privacy and reporting concerns to protect patient anonymity. Less substantive, but relevant to appraisal are more recent tools focusing on “local” evidence, such as Quality Assessment of Community Evidence (QACE) Tools which explicitly incorporate qualitative and more anecdotal sources (80). Furst et al. (65) reviewed tools for assessing context relevant to mental healthcare “ecosystems” (another use of the term from our use here), Their inclusion of patient and regional characteristics would need to be adapted to incorporate eco-social concerns. Moreover, the Social Interventions Research and Evaluation Network (SIREN) produces relevant updates and reviews on tools, which includes for example, the social needs screening tool comparison table to identify the role of housing and workplaces as potential environments. While not explicitly eco-social in nature, these types of tools offer potential to highlight eco-social concerns (81).

Andermann (44) reviewed ways clinical providers could better address specific social determinants of health, including a set of tools for screening individual patients and intervening (e.g., poverty screening tool developed by Center for Effective Practice, undated). Such screening could be built upon with electronic health record templates for some of the exposures and conditions relevant to eco-social concerns (see section Processes above). Analogous to social prescribing approaches (82–84), one can imagine greater use of interventions such as nature prescriptions (85). As well, Andermann (44) urged clinicians to work with other stakeholders and implement tools to assess environments [e.g., Thrive, a US piloted Tool for Health and Resilience in Vulnerable Environments (86)], which includes place determinants such as parks and open spaces, and the state of air, water and soil. Further, the BUILD Healthy Places Network—a large, multi-year funded collaborative explores ways to include diverse and marginalized communities in ways that are generally inclusive of primary care and have a rural primer to guide cross-sector collaborations in ways that are attentive to rural spaces (87). Such assessment can inform group activities addressing eco-social concerns and facilitating opportunities for groups of patients and the broader community (64).

## Implications, Challenges, Opportunities and Questions

By exploring four broad literatures, we uncovered substantial prior work on relevant frameworks, processes and tools, drawing upon different traditions of inquiry and activity. Much will be useful, but others will need to be extended and adapted to incorporate eco-social approaches to health in rural and remote areas. Importantly, our review has several limitations. First, by focusing primarily on the peer-reviewed literature, this review may miss important gray literature contributions, especially pertaining to eco-social approaches to primary care delivery in Indigenous contexts. Second, and relatedly, many of the resources identified were drawn from English-language publications on experiences based in the North American, Oceania, and European contexts, which seems to primarily relate to the nature of health system funding and available published literature. This may present opportunities for future research to learn more specifically about patient- and community-oriented approaches to primary care in other eco-social settings (e.g., Africa, Asia, South America). Third, we did not explicitly review papers for ethical issues arising in the deployment of these frameworks, and future work could examine this to unpack ethical guidance and good conduct practices in deploying the tools and processes uncovered herein. Fourth, our focus was primarily on primary-care approaches, viewed through an eco-social lens. Accordingly, there are massive literatures on more community-oriented approaches leveraging the unique strengths of community development and public health that could add additional nuance and understanding to these issues, but which were ultimately beyond the scope of this review [see for example, (88, 89)].

Nonetheless, our review surfaces a number of challenges that require attention adequately integrate eco-social praxis into primary care practice to promote health in rural and remote areas: complexity, limited durations, additional resources, clinician training, Indigenous perspectives, and governance. Each of these is articulated below in greater detail as an opportunity to promote further research.

*First*, the *complexity* of grappling with both health systems aiming toward greater integration (90) and linkages with other sectors relevant to eco-social concerns, creates challenges for most practitioners and organizations involved (22). The wildly fluctuating drought and flooding cycles, with their huge human health impacts are an example of the complexity of increasing coupling between climate change and human health demanding mitigation measures (91). One unfortunate response is to simplify the complexity, as in environmental assessments of resource development projects which ignore much available social and health data (92). The asynchronous, non-linear process of complex system change can be disorienting, as Strelnick (93) remarked about COPC development. Complex adaptive systems perspectives may be useful to not only guide practitioners through the uncertainties of change, but also to provide some comfort around the incompleteness of any particular transformative effort (94). Expanding to other sectors relevant to ecosystem approaches to health, Waltner-Toews and Kay (95) elegantly laid out various approaches with diverse stakeholders through initial observations-assessment, collaborative learning about both ecological systems-landscapes and societal systems-organizations, and feedback loops during iterative change to improve both landscapes and health. Central to success in this realm is a focus on the *relationships* within the system(s) rather than just the entities within the feedback loops. The non-linear causal loops that characterize complex adaptive systems, although challenging to measure, when seen through an eco-social lens can provide insights and for mutual understanding and more effective joint efforts. This emphasis on complexity and relationships are recognized characteristics of many ecosystem-oriented approaches to health (19, 20).

*Second*, many reports were of demonstration projects or special initiatives which had *limited durations*, of the order of months to years, with only a few reaching decades. In our experience, personal and organizational continuity is a challenge in many rural and remote places, among health care providers, public health practitioners, academics and partner organizations. In particular, better paying jobs in the private resource development industry are often more attractive for those with skill sets that can apply across sectors, just the kind of boundary crossers needed for addressing eco-social concerns (96). Personnel turnover affects organizational memory and relationships, both important for ongoing transformative efforts, and a known challenge in work crossed boundaries among sectors, jurisdictions and mandates (19, 97). In this context primary and public health care could be enhanced by creating organizational information systems that are able to track involvements with communities (similar to individual patient electronic health record systems), relationship-informed handover protocols, and options for continued engagement, even if personnel shift to different organizations, are all required to improve continuity of involvements with communities.

*Third, additional resource requirements* by way of grants or research funding were almost universal across the initiatives in the literature reviewed. These mesh poorly with fee-for-service or even capitation reimbursement models, dominant in primary care financing in many jurisdictions, or with itemized activity-based planning in lean public health organizations. For individual care components, efforts toward patient complexity-based funding could be extended to eco-social concerns, as has been advocated for dealing more effectively with SDH vulnerable patients/community members (36). For community-based components, streams of funding, or collaboration with organizations who have such funding, seems essential (53). However, the role of financing in driving desired service change is probably limited and, in rural attempts specifically, ineffective. Gathering the range of perspectives needed in a collaborative, community-based approach, though often challenging, is often more fruitful. For example, the literature reviewed underscored that academic colleagues' can assist in working with frameworks and data tools, research centers can assist with organizational processes, and universities can facilitate student involvement for documenting processes and contributing to analysis and write-up. A formal commitment to collaborative “tables” bringing together different perspectives at scales from the local to provincial/state levels shows promise (14), especially for avoiding mutual excuse/blame cycles that dissipate both energy and good will when approaching complex issues. “Harm reduction” approaches are informative here, working to “actively engage a diversity of players in finding solutions” in ways that “looks throughout the socio-ecological system at drivers of harm to find strengths, possibilities, and opportunities for solutions in the face of prevailing challenges and uncertainties” [(98). p. 5]. Another example is exemplified by the recent symposium generating ideas on roles health providers can play in “Planning Resilient Communities and Adapting Rural Health Services in British Columbia” (99) and the BC Rural and First Nations Health and Wellness Summit Summary Report (100).

**Figure 1:**
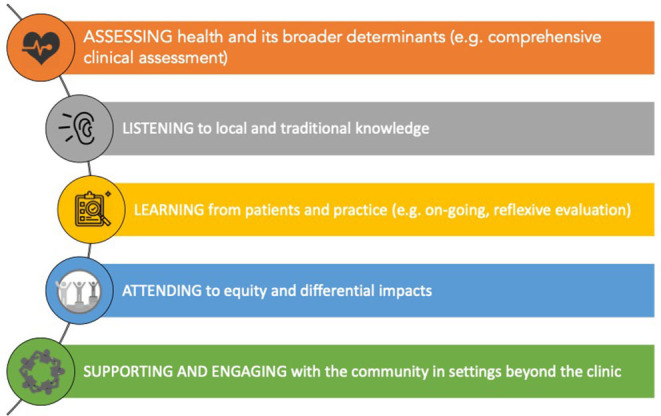
Extending clinical competencies to support the treatment of “communities as patients” in rural and remote places.

*Fourth*, several reports addressed the need for *clinician training* and programs for acculturation into patient-engaged and community-oriented approaches (32). Public health and preventive medicine colleagues have skills in community assessment and population health (101) but these need to be complemented by those among primary care providers. An international movement is emerging focused on transforming health providers' education to build skills relevant to planetary health and sustainable health care (102). An example is the Rural Health Services Research Network of British Columbia's (99) initiative to build on COVID-19 responses and involve primary care providers in promoting community resilience in the face of climate change. We might imagine extending professional competencies to include addressing eco-social concerns, such as modifying the College of Family Physicians of Canada's physician roles and responsibilities to include: (1) ASSESSING health and its broader eco-social determinants (e.g., comprehensive clinical assessment) [Medical Expert role]; (2) LEARNING from patients in practices both individually and as population panels [Professional, Scholar roles]; and (3) SUPPORTING AND ENGAGING with the community-geographical places with other organizations in settings beyond the clinic [Collaborator, Advocate roles] (see Figure 1).

More recent efforts to describe the *Primary Care Home* and *Primary Care Neighborhood* (103) can be seen as pursuing a better understanding of the upstream causes of ill health (i.e., “Why is this patient here in the clinic today” in a more complete sense than previous simple reactivity). At the same time, greater attention to Indigenous ways of seeing, knowing and being has pulled health systems to address *wellness* (100) and a vision of holistic health (100, 104). These developments call primary care toward a greater involvement in population health–and hence on a *bridge* to the realm of community/public health that has been sadly lacking for over a century as clinical care became progressively specialized, technological and disease focused. Some of the more effective examples of such bridging may be found in rural and remote Indigenous communities (100).

*Fifth*, a minority of the literature in colonial contexts e.g., Bourke et al. (1, 33) recognized the growing imperative for primary care and public health systems to be better informed by *Indigenous knowledges and perspectives* in decolonizing research and practice (105). In addition to moving beyond a deficit framing of Indigenous communities in rural and remote regions (9, 106), recognizing the strengths of Indigenous perspectives can overcome false dichotomies between ecological (nature) and social (people) systems. This recognition has perfused recent international calls such as the Association for Medical Education in Europe's consensus statement on Planetary health and education for sustainable healthcare (102, 107, 108). The fact that Indigenous voices and leadership are now being recognized for their importance to informing integrative, eco-social approaches to health (109), has far-reaching implications across primary and public health (78, 106, 110–112), but particularly for eco-social issues such as climate change (113). Those interested in practice that reflects the complex context of health will face increasing imperatives to learn from and with Indigenous-led work as generative pathways to address eco-social concerns in practice in rural and remote contexts (114).

*Sixth*, given the important role of multi-disciplinary teams in our findings, no one practitioner should feel overwhelmed with the learning involved. Professional bodies have set out guidelines on how to develop collaborative care arrangements with social and community supports in a patients' medical neighborhood that could be extended to other sectors (103). The competence to address eco-social concerns will need to be collective (115), distributed across organizations (53) as in social accountability frameworks in primary care (38) and other integrative approaches to working together for health (97). Collective competence involving health and non-health sectors could focus on building *healthy, just* and *sustainable* health systems and societies that are resilient in the face of ecological and social change (22). Yet such multi-sectoral, multi-level involvement demands more explicit attention to *governance*, as noted by practitioner scholars whose work spans health, ecosystems and equity (95, 97, 98) and health geographers focusing on health and health care in rural places (116). Intersectoral partnerships focused on health policy and services in rural areas are evolving in British Columbia through a “Pentagram Partnership Plus” approach involving quarterly meetings and interval consultations with senior public servants responsible for the health care system (100). The question remains: how might governance evolve to support creativity in collaborative work with patients, communities and other sectoral partners to better address eco-social concerns in rural and remote, and resource-dependent contexts?

## Conclusion

Our narrative review of frameworks, processes and tools that can re-imagine and enhance public health and primary care integration to address eco-social health concerns in rural and remote contexts is revealing. While much has been written about the why (e.g., enhancing patient and population health outcomes) and the how (e.g., better intra/inter-organizational collaboration), there was relative little explicit consideration of eco-social approaches to health within these literatures and a potentially problematic tendency to universalize across rural and urban places. Rural, remote and many Indigenous communities face significant pressures when considering the interactions between social and ecological systems. As such, ecological damage is increasingly recognized to exacerbate existing health inequities.

This review identifies the need for more concerted engagement with the combination of ecological decline and ongoing patterns of inequity that need be at the center of increased public health and primary care integration. Mutual goals should include addressing primordial causes of ill-health in rural and remote places, treating patients with humility and in conversation with the places in which they live, work and play, and collectively fostering a sustainable future where health systems are not simply treating the symptoms of ecological decline, but taking an active role in promoting environmental stewardship. This paper outlines hopeful steps in the determined multi-sectoral efforts to change our current “self”-destructive path by broadening the definition of *self* to include the ecosystems on which we depend for our survival.

## Author Contributions

Authorship order reflects contribution to the research, including overall design, analysis, and writing (co-led by CB, SA, and DC), followed by contributions to design, writing, and oversight in alphabetical order (RF, MP, and RW). All authors contributed to the article and approved the submitted version.

## Funding

This work was sponsored in part by a CIHR postdoctoral fellowship held by the lead author (Funding Reference Number: MFE 158126), a Developing Northern Research Collaboration Award provided by the BC SUPPORT Unit of the CIHR Strategy for Patient Oriented Research, and the Northern Health Authority of British Columbia. Collaborative aspects of this work were supported through the collaborative platform of the ECHO Network (Canadian Institute of Health Research Environment and Health Signature Initiative Team Grant [Funding Reference Number: IP4150712]). The funding source had no role in the collection and analysis of data, nor the writing of this manuscript.

## Conflict of Interest

The authors declare that the research was conducted in the absence of any commercial or financial relationships that could be construed as a potential conflict of interest.

## Publisher's Note

All claims expressed in this article are solely those of the authors and do not necessarily represent those of their affiliated organizations, or those of the publisher, the editors and the reviewers. Any product that may be evaluated in this article, or claim that may be made by its manufacturer, is not guaranteed or endorsed by the publisher.

## References

[1] Bourke L Humphreys JS Wakerman J Taylor J. Understanding rural and remote health: a framework for analysis in Australia. Health Place. (2012) 18:496–503. 10.1016/j.healthplace.2012.02.009 22418016

[2] Bourke L Humphreys JS Wakerman J Taylor J. Charting the future course of rural health and remote health in Australia: why we need theory. Aust J Rural Health. (2010) 18:54–8. 10.1111/j.1440-1584.2010.01125.x 20398044

[3] Williams AM Cutchin MP. The rural context of health care provision. J Interprof Care. (2002) 16:107–15. 10.1080/13561820220124120 12028892

[4] Strasser R Neusy AJ. Context counts: training health workers in and for rural and remote areas. Bull World Health Organ. (2010) 88:777–82. 10.2471/BLT.09.072462 20931063 PMC2947041

[5] Hanlon N. Environments of health and care: the contributions of political economy. In: Crooks V Andrews G Pearce J, editors. Handbook of Health Geography. London; New York, NY: Routledge (2018). pp. 88–93.

[6] Hanlon N MacLeod M Reay T Snadden D. Partnering for health care sustainability in smaller urban centers: Why and how a health authority chose to ‘go upstream'. In: Halseth G Markey S Ryser L editors. Service Provision and Rural Sustainability: Infrastructure and Innovation. London; New York, NY: Routledge (2019). pp. 80–94.

[7] Hanlon N Reay T Snadden D MacLeod M. Creating partnerships for health care reform: moving beyond a politics of scale? Int J Health Serv. (2019) 49:51–67. 10.1177/0020731418807094 30335552

[8] Berkes F Folke C Colding J. Linking Social and Ecological Systems: Management Practices and Social Mechanisms for Building Resilience. Cambridge University Press (2000).

[9] Gislason MK Sloan-Morgan V Mitchell-Foster K Parkes MW. Voices from the landscape: storytelling as emergent counter-narratives and collective action from northern BC watersheds. Health Place. (2018) 54:191–9. 10.1016/j.healthplace.2018.08.024 30321859

[10] Schrecker T Birn AE Aguilera MJ. How extractive industries affect health: political economy underpinnings and pathways. Health Place. (2018) 52:135–47. 10.1016/j.healthplace.2018.05.005 29886130

[11] Brisbois B Hoogeveen D Allison S Cole D Fyfe TM Harder HG. Storylines of research on resource extraction and health in Canada: a modified metanarrative synthesis. Soc Sci Med. (2021) 277:113899. 10.1016/j.socscimed.2021.113899 33895709

[12] Fitzpatrick SJ Perkins D Luland T Brown D Corvan E. The effect of context in rural mental health care: understanding integrated services in a small town. Health Place. (2017) 45:70–76. 10.1016/j.healthplace.2017.03.004 28288445

[13] Veitch C. Impact of rurality on environmental determinants and hazards. Aust J Rural Health. (2009) 17:16–20. 10.1111/j.1440-1584.2008.01031.x 19161495

[14] Parkes MW. Cumulative determinants of health impacts in rural, remote, resource-dependent communities. In: Gillingham PM Halseth RG Johnson JC Parkes MW editors. The Integration Imperative: Cumulative Environmental, Community and Health Impacts of Multiple Natural Resource Developments. Cham: Springer International Publishing (2016). pp. 117–49.

[15] Block K Molyneaux R Gibbs L Alkemade N Baker E MacDougall C. The role of the natural environment in disaster recovery: “We live here because we love the bush”. Health Place. (2019) 57:61–9. 10.1016/j.healthplace.2019.03.007 30981069

[16] Lawrence-Bourne J Dalton H Perkins D Farmer J Luscombe G Oelke N. What is rural adversity, how does it affect wellbeing and what are the implications for action? Int J Environ Res Public Health. (2020) 17:7205. 10.3390/ijerph17197205 33019735 PMC7578975

[17] Wakerman J. Innovative rural and remote primary health care models: what do we know and what are the research priorities? Aust J Rural Health. (2009) 17:21–6. 10.1111/j.1440-1584.2008.01032.x 19161496

[18] Canadian Public Health Association (CPHA). Global Change and Public Health: Addressing the Ecological Determinants of Health. Ottawa, ON: Canadian Public Health Association Discussion Paper (2015). Retrieved from: https://www.cpha.ca/discussion-paper-ecologicaldeterminants-health.

[19] Buse CG Oestreiche JS Ellis NS Patrick R Brisbois B Jenkins AP. Public health guide to field developments linking ecosystems, environments and health in the Anthropocene. J Epidemiol Commun Health. (2018) 72:420–5. 10.1136/jech-2017-210082 29330164

[20] Parkes M Allison S Harder H Hoogeveen D Kutzner D Aalhus M. Addressing the environmental, community, and health impacts of resource development: challenges across scales, sectors, and sites. Challenges. (2019) 10:22. 10.3390/challe10010022

[21] Parkes MW Poland B Allison S Cole DC Culbert I Gislason MK. Preparing for the future of public health: ecological determinants of health and the call for an eco-social approach to public health education. Can J Public Health. (2020) 111:60–64. 10.17269/s41997-019-00263-8 31792844 PMC7046913

[22] Buse C Cole DC Parkes MW. Health security in the context of socio-ecological change. Ch 17. In: Lautensach A Lautensach S editors. Human Security in World Affairs: Problems and Opportunities. 2nd ed. A BC Open Campus Publication (2020). Available online at: https://opentextbc.ca/humansecurity/ (accessed August 1, 2021).

[23] Krieger N. Epidemiology and the web of causation: has anyone seen the spider? Soc Sci Med. (1994) 39:887–903. 10.1016/0277-9536(94)90202-X 7992123

[24] Horwitz P Parkes MW. Intertwined strands for ecology in planetary health. Challenges. (2019) 10:20. 10.3390/challe10010020

[25] Krieger N. Theories for social epidemiology in the 21st century: an ecosocial perspective. Int J Epidemiol. (2001) 30:668–77. 10.1093/ije/30.4.668 11511581

[26] Aalhus M. The Social Determinants of Health Impacts of Resource Extraction and Development in Rural and Northern Communities: A Summary of Impacts and Promising Practices for Assessment Monitoring. Northern Health and BC Provincial Health Services Authority 10-420-6106 (WRD 01/18) (2018). Available online at: https://www.northernhealth.ca/sites/northern_health/files/services/office-health-resource-development/documents/impacts-promising-practices-assessment-monitoring.pdf (accessed January 2, 2020).

[27] Buse C Lai V Cornish K Parkes M. Towards environmental health equity in health impact assessment: innovations and opportunities. Int J Public Health. (2019) 64:15–26. 10.1007/s00038-018-1135-1 29911285

[28] Longlett SK Kruse JE Wesley RM. Community-oriented primary care: historical perspective. J Am Board Fam Pract. (2001) 14:54–63. 11206694

[29] The Folsom Group. Communities of solution: the Folsom Report revisited. Ann Fam Med. (2012) 10:250–60. 10.1370/afm.1350 22585890 PMC3354975

[30] Gofin J Gofin R. Community oriented primary care: a public health model in primary care. Pan Am J Public Health. (2007) 21:1–12. 10.1590/s1020-49892007000200012 17565804

[31] Gofin J Foz G. Training and Application of Community-oriented Primary Care (COPC) through family medicine in Catalonia, Spain. Int Fam Med. (2008) 40:196–202. 18320398

[32] Blumenthal D. Clinical community health: revisiting “the community as patient”. Educ Health. (2009) 22:234. 20029748 PMC4527153

[33] Bourke L Humphreys JS Wakerman J Taylor J. Understanding drivers of rural and remote health outcomes: a conceptual framework in action. Aust J Rural Health. (2012) 20:318–23. 10.1111/j.1440-1584.2012.01312.x 23181816

[34] Bodenheimer T Sinsky C. From triple to quadruple aim: care of the patient requires care of the provider. Ann Fam Med. (2014) 12:573–6. 10.1370/afm.1713 25384822 PMC4226781

[35] Miranda ML Ferranti J Strauss B Neelon B Califf RM. Geographic health information systems: a platform to support the ‘triple aim'. Health Aff. (2013) 32:1608–15. 10.1377/hlthaff.2012.1199 24019366 PMC4076782

[36] Tipirneni R Vickery KD Ehlinger EP. Accountable communities for health: moving from providing accountable care to creating health. Ann Fam Med. (2015) 13:367–9. 10.1370/afm.1813 26195684 PMC4508180

[37] Pelletier F Lesage A Boisvert C Denis F Bonin J Kisely S. Feasibility and acceptability of patient partnership to improve access to primary care for the physical health of patients with severe mental illnesses: an interactive guide. Int J Equity Health. (2015) 14:1–9. 10.1186/s12939-015-0200-0 26370926 PMC4568580

[38] Woollard RF Buchman S Meili R Strasser R Alexander I Goel R. Social accountability at the meso level: Into the community. Can Fam. (2016) 62:538–40. 27412198 PMC4955073

[39] Holroyd-Leduc JR Resin J Ashley L Barwich D Elliott J Huras P. Giving voice to older adults living with frailty and their family caregivers: engagement of older adults living with frailty in research, health care decision making, and in health policy. Res Involv Engag. (2016) 2:1–19. 10.1186/s40900-016-0038-7 29062523 PMC5611602

[40] Orkin AM Bharmal A Cram J Kouyoumdjian FG Pinto AD Upshur R. Clinical population medicine: integrating clinical medicine and population health in practice. Ann Fam Med. (2017) 15:405–9. 10.1370/afm.2143 28893808 PMC5593721

[41] Kaufman A. Theory vs practice: should primary care practice take on social determinants of health now? yes. Ann Fam Med. (2016) 14:100-1. 10.1370/afm.1915 https://dx.doi.org/10.1370 26951582 PMC4781510

[42] Martin-Misener R Valaitis R Wong ST MacDonald M Meagher-Stewart D Kaczorowski J. A scoping literature review of collaboration between primary care and public health. Prim Health Cae Res Develop. (2012) 13:327-46. 10.1017/S1463423611000491 22353204

[43] Shahzad M Upshur R Donnelly P Bharmal A Wei X Feng P. A population-based approach to integrated healthcare delivery: a scoping review of clinical care and public health collaboration. BMC Public Health. (2019) 19:708. 10.1186/s12889-019-7002-z 31174501 PMC6556001

[44] Andermann A. Taking action on the social determinants of health in clinical practice: a framework for health professionals. Can Med Assoc J. (2016) 188:E474–83. 10.1503/cmaj.160177 27503870 PMC5135524

[45] Pope C Ziebland S Mays N. Qualitative research in health care: analysing qualitative data. Br Med J. (2000) 320:114–6. 10.1136/bmj.320.7227.114 10625273 PMC1117368

[46] Leonhardt KK Bonin D Pagel P. Partners in safety: implementing a community-based patient safety advisory council. Wisconsin Med J. (2006) 105:54–60. 17256713

[47] Tisnado DM Sablan-Santos L Guevara L Quitugua L Castro K Aromin J. A case study in Chamorro community and academic engagement for a community-partnered research approach. California J Health Promot. (2010) 8:39–51. 10.32398/cjhp.v8iSI.2041 26726298 PMC4696771

[48] Joosten YA Israel TL Williams NA Boone LR Schlundt DG Mouton CP. Community engagement studios: a structured approach to obtaining meaningful input from stakeholders to inform research. Acad Med. (2015) 90:1646–50. 10.1097/ACM.0000000000000794 26107879 PMC4654264

[49] Joosten YA Israel TL Head A Vaughn Y Gil VV Mouton C. Enhancing translational researchers' ability to collaborate with community stakeholders: lessons from the Community Engagement Studio. J Clin Transl Sci. (2018) 2:201–7. 10.1017/cts.2018.323 30820357 PMC6382358

[50] Etchegary H Bishop L Street C Aubrey-Bassler K Humphries D Vat LE. Engaging patients in health research: identifying research priorities through community town halls. BMC Health Serv Res. (2017) 17:1–7. 10.1186/s12913-017-2138-y 28284232 PMC5346234

[51] Marcus TS Hugo J Jinabhai CC. Which primary care model? A qualitative analysis of ward-based outreach teams in South Africa. Afr J Prim Health Care Fam Med. (2017) 9:1–8. 10.4102/phcfm.v9i1.1252 28582994 PMC5458565

[52] Moosa S Derese A Peersman W. Insights of health district managers on the implementation of primary health care outreach teams in Johannesburg, South Africa: a descriptive study with focus group discussions. Hum Resour Health. (2017) 15:2–9. 10.1186/s12960-017-0183-6 28109275 PMC5251300

[53] Kaufman AW Dickinson P Fagnan LJ Duffy FD Parchman ML Rhyne RL. The role of health extension in practice transformation and community health improvement: lessons from 5 case studies. Ann Fam Med. (2019) 17:S67–72. 10.1370/afm.2409 31405879 PMC6827669

[54] Gosling R Davies S Hussey J. How integrating primary care and public health could improve population health outcomes: a view from Liverpool, UK. Public Health Res Pract. (2016) 26:e2611602. 10.17061/phrp2611602 26863165

[55] Reid CE Brauer M Johnston FH Jerrett M Balmes JR Elliott CT. Critical review of health impacts of wildfire smoke exposure. Environ Health Perspect. (2016) 124:1334–43. 10.1289/ehp.1409277 27082891 PMC5010409

[56] Calman N Hauser D Lurio J Wu W Pichardo M. Strengthening public health and primary care collaboration through electronic health records. Am J Public Health. (2012) 102:e13–18. 10.2105/AJPH.2012.301000 22994274 PMC3477979

[57] Bruckner J Barr B. Data-driven population health. N C Med J. (2014) 75:200–201. 10.18043/ncm.75.3.200 24830496

[58] Johnston SJ Belanger E Wong K Snadden D. How can rural community-engaged health services planning affect sustainable health care system changes? BMJ Open. (2021) 11:e047165. 10.1136/bmjopen-2020-047165 34649845 PMC8522661

[59] Mullan F Phillips RL Kinman EL. Geographic retrofitting: a method of community definition in community-oriented primary care practices. Fam Med. (2004) 36:440–6. 15181557

[60] Dulin MF Ludden TM Tapp H Blackwell J de Hernandez BU Smith HA. Using Geographic Information Systems (GIS) to understand a community's primary care needs. J Am Board Fam Med. (2010) 23:13–21. 10.3122/jabfm.2010.01.090135 20051538

[61] Lebrun LA Shi L Chowdhury J Sripipatana A Zhu J Sharma R. Primary care and public health activities in select U.S. Health Centers: documenting successes, barriers, lessons learned. Am J Prev Med. (2012) 42(Suppl. 2):S191–202. 10.1016/j.amepre.2012.03.011 22704437

[62] Lewis JH Whelihan K Navarro I Boyle KR SDH Card Study Implementation Team. Community health center provider ability to identify, treat and account for the social determinants of health: a card study. BMC Fam Pract. (2016) 17:1–12. 10.1186/s12875-016-0526-8 27567892 PMC5002327

[63] Gold R Bunce A Cowburn S Dambrun K Dearing M Middendorf M. Adoption of social determinants of health EHR tools by community health centers. Ann Fam Med. (2018) 16:399–407. 10.1370/afm.2275 30201636 PMC6131002

[64] Young P Cole DC Wong Labow C Thompson C. Addressing the Ecological Determinants of Health (EDH): Contributions of Canadian CHCs. (2018). Available online at: https://www.allianceon.org/research/Addressing-ecological-determinants-health-EDH-contributions-Canadian-CHCs-until-2018

[65] Furst MA Gandré C López-Alberca CR Salvador-Carulla L. Healthcare ecosystems research in mental health: a scoping review of methods to describe the context of local care delivery. BMC Health Serv Res. (2016) 19:1–13. 10.1186/s12913-019-4005-5 30885186 PMC6423877

[66] Mullan F Epstein L. Community-oriented primary care: new relevance in a changing world. Am J Public Health. (2002) 92:1748-55. 10.2105/ajph.92.11.1748 12406800 PMC3221479

[67] Gavagan T. A systematic review of COPC: evidence for effectiveness. J Health Care Poor Underserved. (2008) 19:963–80. 10.1353/hpu.0.0061 18677083

[68] National National Academies of Sciences, Engineering, and and Medicine *Implementing High-Quality Primary Care: Rebuilding the Foundation of Health Care*. Washington, DC: The National Academies Press (2021). 34251766

[69] Committee Committee on Integrating Primary Care and Public Health Board on Population, Health, Public Health. Practice, Institute of Medicine. Primary Care and Public Health: Exploring Integration to Improve Population Health. Washington, DC: National Academies Press (US) (2012). 24851288

[70] Sprague JB Koo D Castrucci BC Michener JL. The Practical Playbook : Public Health and Primary Care Together. New York, NY: Oxford University Press (2016).

[71] World Health Organization. Primary Health Care: Closing the Gap Between Public Health and Primary Care Through Integration. Geneva: WHO (2019). Available online at: https://apps.who.int/iris/rest/bitstreams/1242113/retrieve

[72] Nilson C. Collaborative risk-driven intervention: research supporting technology-enabled opportunities for upstream virtual services in rural and remote communities. J Commun Saf Well Being. (2017) 2:76–86. 10.35502/jcswb.55

[73] Fleming MD Shim JK Yen IH Thompson-Lastad A Rubin S Van Natta M. Patient engagement at the margins: health care providers' assessments of engagement and the structural determinants of health in the safety-net. Soc Sci Med. (2017) 183:11–18. 10.1016/j.socscimed.2017.04.028 28445806 PMC5654327

[74] Munoz SA Bradley S. We've got what the NHS ultimately intended for us: experiences of community engagement in rural primary care services change. Soc Sci Med. (2021) 280:114033. 10.1016/j.socscimed.2021.114033 34044185

[75] Farmer J Currie M Kenny A Munoz SA. An exploration of the longer-term impacts of community participation in rural health services design. Soc Sci Med. (2015) 141:64–71. 10.1016/j.socscimed.2015.07.021 26248306

[76] Cunsolo A Ellis NE. Ecological grief as a mental health response to climate change-related loss. Nat Clim Chang. (2018) 8:275–81. 10.1038/s41558-018-0092-2

[77] Bondy M Cole DC. Change as a double edged sword: ecological farmers' stressors and health with changes in farming in Grey County, Ontario. J Rural Commun Dev. (2019) 14:114–31.

[78] Redvers J. “The land is a healer”: perspectives on land-based healing from Indigenous practitioners in northern Canada. Int J Indigenous Health. (2020) 15:90–107. 10.32799/ijih.v15i1.34046

[79] First Nations Health Authority. The BC Local Environmental Observer (LEO) Network. Available online at: https://www.fnha.ca/AboutSite/NewsAndEventsSite/NewsSite/Documents/The-Local-Environmental-Observer-LEO-Network.pdf (August 2, 2021)

[80] National Collaborating Centre for Methods Tools (NCCMT). User's Guide: Quality Assessment of Community Evidence (QACE) Tools and QACE Tool A: Quality Assessment of Evidence for Local Health Issues, Local Context. (2020). Available online at: https://www.nccmt.ca/qace (accessed February 3, 2022).

[81] SIREN. Social Needs Screening Tool Comparison Table. San Francisco, CA: USFC (2019). Available online at: https://sirenetwork.ucsf.edu/tools-resources/resources/screening-tools-comparison

[82] Kilgarriff-Foster A O'Cathain A. Exploring the components and impact of social prescribing. J Public Ment Health. (2015) 14:127–34. 10.1108/JPMH-06-2014-0027

[83] Alliance for Healthy Communities. Rx: Community - Social Prescribing in Ontario. Available online at: https://www.allianceon.org/Rx-Community-Social-Prescribing-Ontario (accessed June 3, 2021).

[84] Bhatti S Rayner J Pinto AD Mulligan K Cole DC. Using self-determination theory to understand the social prescribing process: a qualitative study. Br J Gen Pract Open. (2021) 5:BJGPO.2020.0153. 10.3399/BJGPO.2020.0153 33402331 PMC8170608

[85] Hunter MR Gillespie BW Chen SY-P. Urban nature experiences reduce stress in the context of daily life based on salivary biomarkers. Front Psychol. (2019) 10:1–16. 10.3389/fpsyg.2019.00722 31019479 PMC6458297

[86] Prevention Institute. THRIVE: Tool for Health and Resilience in Vulnerable Environments. Available online at: https://www.preventioninstitute.org/tools/thrive-tool-health-resilience-vulnerable-environments

[87] Build Healthy Places Network. A *Primer for Multi-Sector Health Partnerships in Rural Areas and Small Cities*. (2022). Available online at: https://buildhealthyplaces.org/tools-resources/rural-primer/ (accessed February 3, 2022).

[88] Department of Health and Human Services. Principles of Community Engagement. 2nd ed. Clinical and Translational Science Awards Consortium Community Engagement Key Function Committee Task Force on the Principles of Community Engagement. NIH Publication No. 11-7782 (2011). pp. 194. Available online at: https://www.atsdr.cdc.gov/communityengagement/pdf/PCE_Report_508_FINAL.pdf

[89] Organizing Committee for Assessing Meaningful Community Engagemetn in Health and Healthcare Policies Programs. National Academy of Medicine: Perspectives. (2022). pp. 12. Available online at: https://nam.edu/wp-content/uploads/2022/02/Assessing-Meaningful-Community-Engagement.pdf

[90] Tsasis P Evans JM Owen S. Reframing the challenges to integrated care: a complex-adaptive systems perspective. Int J Integr Care. (2012) 12:1–11. 10.5334/ijic.843 23593051 PMC3601537

[91] Sudmeier-Rieux K Ash N Murti R. Environmental Guidance Note for Disaster Risk Reduction: Healthy Ecosystems for Human Security and Climate Change Adaptation. 2013 ed. Gland: IUCN. (2013). pp. iii + 34. Available online at: https://www.iucn.org/sites/dev/files/content/documents/2013_iucn_bookv2.pdf

[92] Buse CG Cornisk K Parkes MW Harder H Fumerton R Rasali D. Towards More Robust and Locally Meaningful Indicators for Monitoring the Social Determinants of Health Related to Resource Development Across Northern BC. Report prepared for Northern Health. Prince George, BC: University of Northern British Columbia (2018). Available online at: https://www.northernhealth.ca/sites/northern_health/files/services/office-health-resource-development/documents/nh-unbc-indicators-report.pdf

[93] Strelnick AH. Community-oriented primary care: the state of an art. Arch Fam Med. (1999) 8:550–2. 10.1001/archfami.8.6.550 10575397

[94] Sturmberg JP O'Halloran DM Martin CM. Understanding health system reform – a complex adaptive systems perspective. J Eval Clin Pract. (2012) 18:202–8. 10.1111/j.1365-2753.2011.01792.x 22221420

[95] Waltner-Toews D Kay J. The evolution of an ecosystem approach: the diamond schematic and an adaptive methodology for ecosystem sustainability and health. Ecol Soc. (2005) 10:38. 10.5751/ES-01214-100138

[96] Kilpatrick S Cheers B Gilles M Taylor J. Boundary crossers, communities, and health: exploring the role of rural health professionals. Health Place. (2009) 15:284–90. 10.1016/j.healthplace.2008.05.008 18617433

[97] Parkes MW. Working together for WHOLE systems: approaching well-being and health, while oriented to living-systems and equity. In: Stephen C, editor. Animals, Health and Society: Health Promotion, Harm Reduction, and Health Equity in a One Health World. Boca Raton, FL: CRC Press (2021). pp. 71–94.

[98] Stephen C Wittrock J Wade J. Using a harm reduction approach in an environmental case study of fish and wildlife health. EcoHealth. (2018) 15:4–7. 10.1007/s10393-017-1311-4 29362965

[99] Rural Health Services Research Network of British Columbia (RHSRNbc). Planning resilient communities and adapting rural health services in British Columbia. In: December 2020 Symposium Proceedings. (2020). pp. 14. Available online at: https://med-fom-rhsrnbc.sites.olt.ubc.ca/files/2021/01/Planning-Resilient-Communities-and-Adapting-Rural-Health-Services-in-British-Columbia-6.pdf

[100] Rural Coordinating Centre of British Columbia (RCCbc). BC *Rural and First Nations Health and Wellness Summit Summary Report*. (2020). Available online at: https://rccbc.ca/wp-content/uploads/2020/08/BC-Rural-and-First-Nations-Health-and-Wellness-Summit-Summary-Report.pdf

[101] Peik SM Mohan KM Baba T Donadel M Labruto A Loh LC. Comparison of public health and preventive medicine physician specialty training in six countries: identifying challenges and opportunities. Med Teach. (2016) 38:1146–51. 10.3109/0142159X.2016.1170784 27093229

[102] McKimm J Redvers N El Omrani O Parkes MW Elf M Woollard R. Education for sustainable healthcare: leadership to get from here to there. Med Teach. (2020) 10:1123–7. 10.1080/0142159X.2020.1795104 32776858

[103] College of Family Physicians of Canada (CFPC). Best Advice Guide: Patient's Medical Neighbourhood. Mississauga, ON: College of Family Physicians of Canada (2020). p. 15. Available online at: https://patientsmedicalhome.ca/resources/best-advice-guides/the-patients-medical-neighbourhood/

[104] Durkalec A Furgal C Skinner MW Sheldon T. Climate change influences on environment as a determinant of Indigenous health: relationships to place, sea ice, and health in an Inuit community. Soc Sci Med. (2015) 136:17–26. 10.1016/j.socscimed.2015.04.026 25974138

[105] Sasakamoose J Bellegarde T Sutherland W Pete S McKay-McNabb K. Miýo-pimātisiwin Developing Indigenous Cultural Responsiveness Theory (ICRT): improving Indigenous health and well-being. Int Indigenous Policy J. (2017) 8:4. 10.18584/iipj.2017.8.4.1

[106] Aldred TL Alderfer-Mumma C de Leeuw S Farrales M Greenwood M Hoogeveen D. Mining sick: creatively unsettling normative narratives about industry, environment, extraction, and the health geographies of rural, remote, northern, and Indigenous communities in British Columbia. Can Geogr. (2020) 65:82–96. 10.1111/cag.12660 33888912 PMC8049089

[107] Shaw E Walpole S McLean M Alvarez-Nieto C Barna S Bazin K. AMEE Consensus Statement: Planetary health and education for sustainable healthcare. Med Teach. (2021) 43:272–86. 10.1080/0142159X.2020.1860207 33602043

[108] Redvers N Schultz C Prince MV Cunnigham M Jones R Blondin B. Indigenous perspectives on education for sustainable healthcare. Med Teach. (2020) 42:1085–90. 10.1080/0142159X.2020.1791320 32657230

[109] Waddell CM de Jager MD Gobeil J. Healing journeys: indigenous men's reflections on resources and barriers to mental wellness. Soc Sci Med. (2021) 270:113696. 10.1016/j.socscimed.2021.113696 33465597

[110] Ratima M Martin D Castleden H Delormier T. Indigenous voices and knowledge systems – promoting planetary health, health equity, and sustainable development now and for future generations. Glob Health Promot. (2019) 26:3–5. 10.1177/1757975919838487 30964406

[111] Redvers N Poelina A Schultz C Kobei DM Githaiga C Perdrisat M. Indigenous natural and first law in planetary health. Challenges. (2020) 11:29. 10.3390/challe11020029

[112] Waters S. The environment: the ecosystems is our health system. In: National Collaborating Centre for Indigenous Health (NCCIH). Visioning the Future: First Nations, Inuit, and Métis Population and Public Health. National Collaborating Centre for Indigenous (2021). p. 43–5. Available online at: https://www.nccih.ca/495/Visioning_the_Future__First_Nations,_Inuit,___M%C3%A9tis_Population_and_Public_Health_.nccih?id=10351

[113] National Collaborating Centre for Indigenous Health (NCCIH). Climate change and indigenous peoples' health in Canada. In: Berry P Schnitter R editors. Health of Canadians in a Changing Climate: Advancing our Knowledge for Action. Ottawa, ON: Government of Canada (2022). Available online at: https://changingclimate.ca/health-in-a-changing-climate/chapter/2-0/

[114] National Collaborating Centre for Indigenous Health (NCCIH). Visioning the Future: First Nations, Inuit, and Métis Population and Public Health. National Collaborating Centre for Indigenous Health (2021). Available online at: https://www.nccih.ca/495/Visioning_the_Future__First_Nations,_Inuit,___M%C3%A9tis_Population_and_Public_Health_.nccih?id=10351

[115] Lingard L. What we see and don't see when we look at ‘competence': notes on a god term. Adv Health Sci Educ. (2009) 14:625–8. 10.1007/s10459-009-9206-y 19856124

[116] Herron R Skinner M. Rural places and spaces of health and health care. Ch 38. In: Crooks VA Gavin Andrews JGA Pearce J editors. Routledge Handbook of Health Geography. New York, NY: Routledge (2018). pp. 267–72.

